# Exploring the relationship between urban visual density and responsible tourism behavior: a multimodal study of Macao

**DOI:** 10.3389/fpsyg.2026.1783451

**Published:** 2026-03-26

**Authors:** Youcheng Wang, Haoran Lyu, Yixi Wu, Yifei Ouyang

**Affiliations:** 1 Faculty of International Tourism and Management, City University of Macau, Macao, Macao SAR, China; 2 Institute of Urban and Sustainable Development, City University of Macau, Macao, Macao SAR, China; 3 Department of Art Design, Nanfang College, Guangzhou, China

**Keywords:** high-density urban destination, Macao, multimodal analysis, responsible tourism behavior, street-level visual indicators, urban visual density

## Abstract

**Introduction:**

Understanding responsible tourism behavior in high-density urban destinations is critical for sustainable tourism development. However, limited research has examined how tourists’ psychological evaluations are situated within objectively measured urban visual environments.

**Methods:**

This study adopted a multimodal design combining survey-based behavioral modeling and street-level visual analysis in Macao. Survey data from 519 non-local visitors were analyzed using covariance-based structural equation modeling to examine relationships among destination image, perceived value, perceived risk, satisfaction, attitude, and responsible tourism behavioral intention. Street-level visual indicators, including the Green View Index, Sky View Factor, and Building Enclosure Index, were derived from street view imagery using deep learning–based semantic segmentation and summarized at the functional zone level.

**Results:**

Destination image, perceived value, and satisfaction were positively associated with attitudes toward responsible tourism behavior, whereas perceived risk is negatively associated with satisfaction and attitude. A zone-level descriptive comparison further suggested that visually open and green environments tended to coincide with more favorable destination evaluations, while visually enclosed environments aligned with higher perceived risk.

**Discussion:**

These findings highlight the importance of environmental context in understanding responsible tourism behavior in high-density urban destinations. The observed relationships should be interpreted as contextual alignment rather than evidence of independent environmental effects or formal cross-zone causal inference.

## 1 Introduction

The continuous expansion of global tourism has placed increasing pressure on natural resources, cultural heritage, and urban environments, raising concerns about the long-term sustainability of tourism development. As tourism activities intensify, especially in urban settings, destinations face challenges related to overcrowding, environmental degradation, and declining visitor experience quality. In response to these issues, promoting responsible tourism behavior—defined as tourists’ voluntary actions aimed at minimizing negative environmental and social impacts—has become a central objective in sustainable tourism research and destination management (Mihalic, 2020; Gössling and Scott, 2022).

In this study, the focus is placed on visual environmental density at the street level, referring to perceptible spatial enclosure, visual openness, and the presence of visible greenery. This concept differs from conventional measures of urban density, such as population density or development intensity. Instead, it aligns more closely with literature on visual landscape assessment and streetscape perception, which emphasizes experiential attributes including enclosure, openness, coherence, and complexity (Ode et al., 2008; Tveit et al., 2006; Gobster et al., 2007). By clarifying this distinction, urban visual density in this study is conceptualized as a perceptual attribute rather than a demographic or purely morphological indicator. Prior research on responsible tourism behavior has been conducted across diverse spatial contexts, including island destinations and nature-based tourism areas, where environmental stewardship motivations are often emphasized (Han et al., 2010; Lee, 2011), as well as urban and heritage cities, where spatial density, congestion, and perceived risk tend to play a more prominent role in shaping visitor evaluations (Bae and Chang, 2021; Rasoolimanesh et al., 2017). These variations suggest that responsible tourism behavior is context-sensitive and influenced by environmental and spatial conditions specific to different destination types (Afshardoost and Eshghi, 2020). This contextual differentiation underscores the importance of examining responsible tourism behavior within high-density urban morphological settings.

Macao provides a distinctive empirical context for examining these issues. Despite its small land area, Macao accommodates exceptionally high tourist volumes and exhibits one of the highest population densities globally. Its tourism landscape is characterized by a pronounced dual structure, in which a UNESCO-listed historic center coexists with large-scale gaming and entertainment districts dominated by high-rise and highly engineered urban forms (McCartney, 2020). This duality generates substantial spatial heterogeneity across the city, ranging from vertically enclosed heritage streets to visually expansive but intensively developed resort areas. Such environmental contrasts may coincide with differences in tourists’ perceptions of comfort, crowding, and environmental quality, thereby shaping satisfaction and attitudinal evaluation (Wan and Li, 2013).

Research on responsible tourism behavior has frequently drawn on the Theory of Planned Behavior, which conceptualizes behavioral intention as shaped by attitudes, subjective norms, and perceived behavioral control (Ajzen, 1991). In tourism contexts, however, empirical models often extend the theory by incorporating destination-related experiential evaluations that precede attitude formation, such as destination image, perceived value, satisfaction, and perceived risk (Han, 2015; Ulker-Demirel and Ciftci, 2020). Building on this practice, the present study focuses on the attitudinal pathway and models these experiential constructs as antecedents of attitude and responsible tourism behavioral intention. Subjective norms and perceived behavioral control are not explicitly included, and the framework is therefore positioned as a context-specific attitudinal extension rather than a full replication of the original Theory of Planned Behavior. This positioning allows the model to better reflect how tourists’ evaluations formed in high-density urban settings translate into intention, while keeping theoretical claims appropriately bounded.

Recent advances in urban informatics and computer vision offer new methodological opportunities to address this gap. Street view imagery (SVI), combined with deep learning–based semantic segmentation, enables the objective measurement of visual environmental characteristics at a fine spatial scale. Indicators such as the Green View Index and Sky View Factor have been increasingly used to capture aspects of urban form and visual openness that are closely related to human spatial experience. Despite their growing application in urban studies, these methods have rarely been integrated into tourism behavior research, and empirical evidence linking objectively measured visual environments with tourists’ psychological evaluations and responsible behavioral intentions remains limited.

To address these limitations, this study adopts a multimodal research design that integrates survey-based behavioral modeling with objective measurement of the street-level visual environment. Drawing on the Theory of Planned Behavior, the study examines relationships among destination image, perceived value, perceived risk, satisfaction, attitude, and responsible tourism behavioral intention using survey data collected from tourists in Macao. Importantly, the visual indicators are not incorporated into the structural equation model as individual-level predictors. Instead, street view–derived metrics are aggregated at the functional zone level to characterize overall visual conditions. By aligning zone-level visual characteristics with differences in latent psychological evaluations, the analysis provides contextual interpretation rather than causal estimation. In this design, the visual environment is treated as an experiential context rather than as a direct determinant of individual behavioral intention.

Rather than incorporating visual indicators directly into behavioral prediction models, this study uses street view–derived visual metrics to contextualize tourists’ psychological evaluations across visually distinct urban environments. By aligning subjective evaluations with aggregated visual characteristics at the functional zone level, the analysis examines whether spatial variation in destination evaluations and perceived risk corresponds to differences in visual openness, greenery, and building enclosure. In doing so, the study positions the visual environment as an experiential context, rather than as a direct determinant of individual behavior.

## 2 Data and methods

### 2.1 Study area and spatial zoning

This study was conducted in Macao, a high-density urban tourism destination located on the western bank of the Pearl River Delta. With a total land area of approximately 33.3 km^2^, Macao receives a large volume of tourists annually, particularly from Mainland China and Hong Kong, which places substantial pressure on its urban infrastructure and public space (McCartney, 2020). The city’s tourism landscape is characterized by a pronounced dual structure, combining heritage conservation with large-scale gaming and entertainment development, making it a suitable setting for examining responsible tourism behavior in dense urban environments.

To capture spatial heterogeneity in the tourism environment, the study area was divided into three functional zones based on land use patterns and dominant tourism activities. Zone A (Historic Heritage Zone) is centered on the UNESCO-listed Historic Centre of Macao and is characterized by cultural landmarks, narrow streets, and dense historical fabric. Zone B (Modern Entertainment Zone) is dominated by integrated resorts and large-scale entertainment complexes featuring high-rise buildings and intensively engineered urban forms. Zone C (Coastal and Natural Zone) consists primarily of open spaces, waterfront areas, and natural landscapes with comparatively lower building density. This zoning strategy allows for systematic comparison across environments with distinct visual and spatial characteristics and has been widely adopted in urban tourism research to capture intra-city variation (Liu et al., 2021; Wan and Li, 2013).

**Figure 1:**
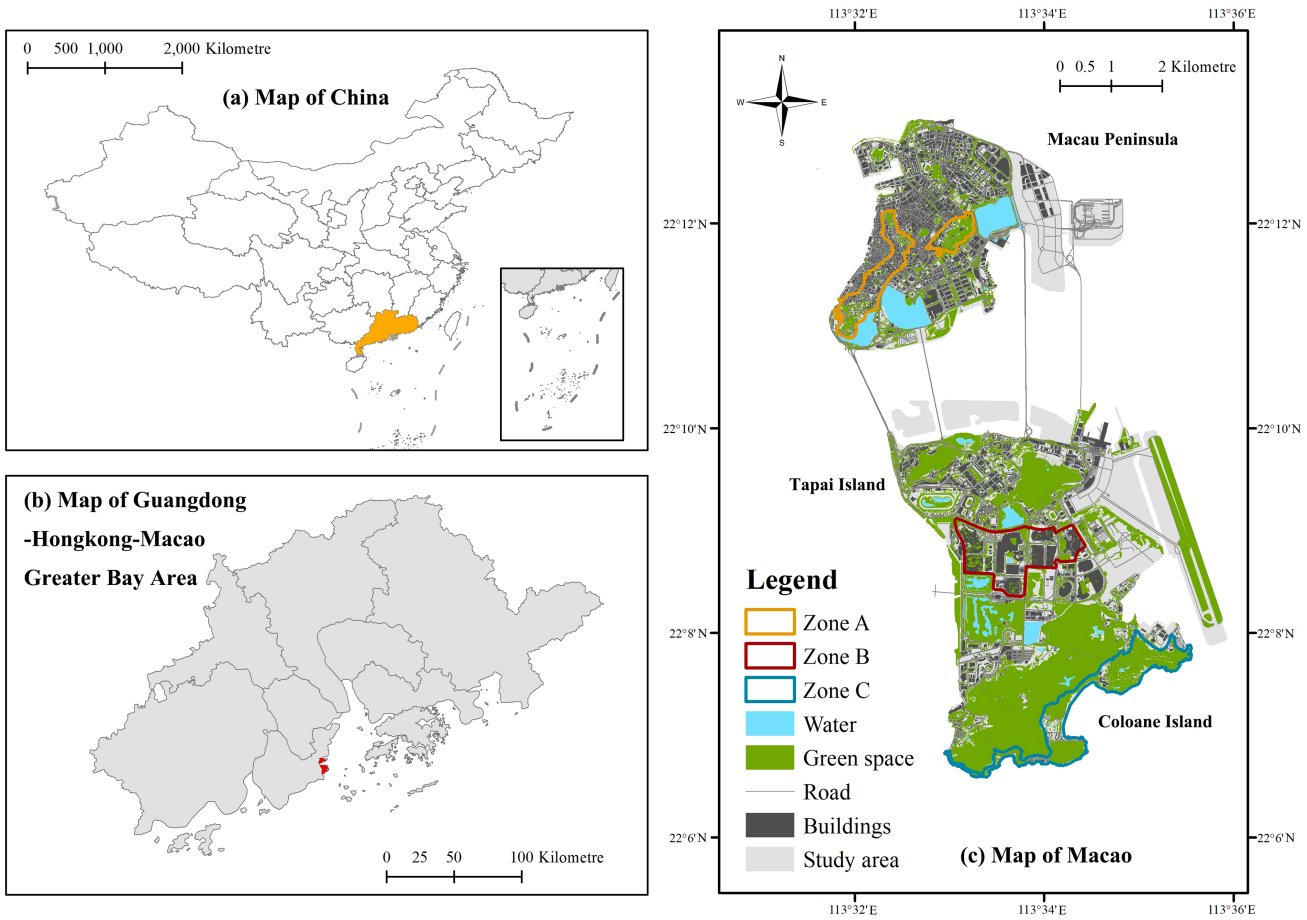
Geographical location and functional zoning of the study area in Macao SAR. **(a)** Map of China; **(b)** map of Guangdong–Hong Kong–Macao Greater Bay Area; **(c)** map of Macau: Zone A: historic heritage zone; Zone B: modern entertainment zone; Zone C: coastal & natural zone.

**Figure 2:**
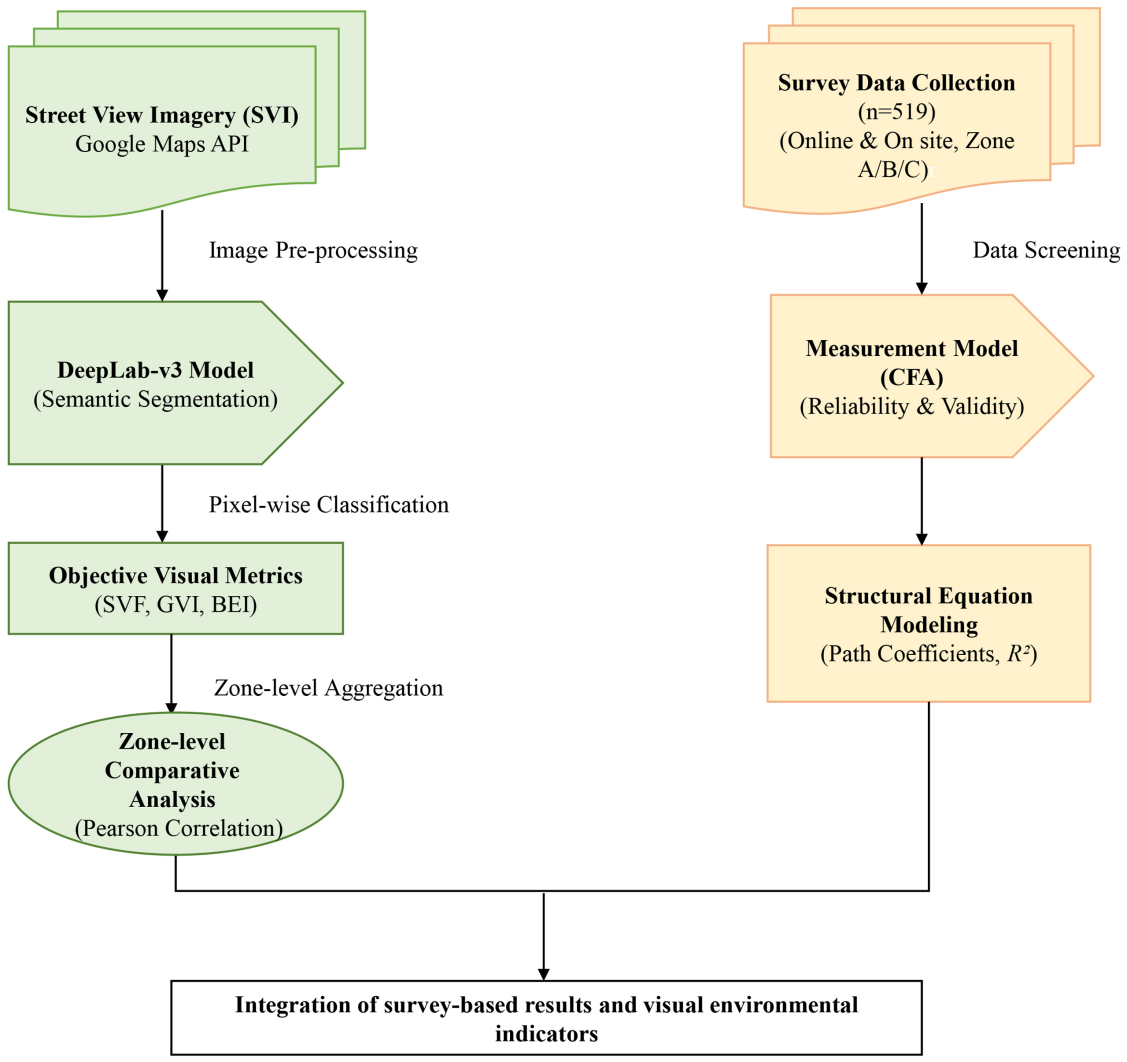
Technical workflow of multimodal data processing and analysis.

By distinguishing zones with contrasting physical environments, the spatial framework facilitates analysis of how tourists’ psychological evaluations and behaviors vary across different urban contexts. The geographical location of Macao and the functional zoning scheme are shown in Figure 1. To provide a comprehensive overview of the research design, the technical workflow—illustrating the integration of deep learning-based visual quantification and survey data analysis—is presented in Figure 2.

### 2.2 Survey data collection

To examine tourists’ psychological evaluations and responsible behavioral intentions, a structured questionnaire was developed based on established and validated measurement scales in the tourism literature. The survey instrument measured six latent constructs: destination image, perceived value, perceived risk, satisfaction, attitude, and responsible tourism behavioral intention. In the present study, perceived risk is conceptualized as a context-specific form of environmental and spatial risk within high-density urban tourism settings. The construct captures concerns related to congestion, crowding discomfort, and spatial complexity (e.g., difficulty navigating dense street networks), rather than broader financial, health-related, or destination-level tourism risks commonly discussed in the tourism literature. All measurement items were originally developed in English and translated into Chinese using a standard translation and back-translation procedure conducted by bilingual researchers to ensure semantic equivalence. Responses were recorded using a five-point Likert scale ranging from 1 (“strongly disagree”) to 5 (“strongly agree”) (Likert, 1932). Prior to the main survey, a pilot test with 30 respondents was conducted to refine item wording and assess content validity (Churchill, 1979).

Data collection was carried out over a twelve-month period from December 2023 to December 2024. A dual-channel sampling strategy was employed to reduce coverage bias and enhance sample diversity (Dillman et al., 2014). Online questionnaires were distributed through major social media platforms in China, including WeChat and Xiaohongshu, targeting post-trip visitors in order to capture reflective evaluations of their travel experiences (Buhalis and Law, 2008). To ensure spatial correspondence with the objective visual data, online respondents were required to identify the zone (Zone A, B, or C) in which they spent most of their time during their visit. Only respondents who could clearly identify a primary activity zone were included in the analysis. As online responses rely on retrospective self-report, the possibility of recall bias cannot be fully excluded.

**Figure 3:**
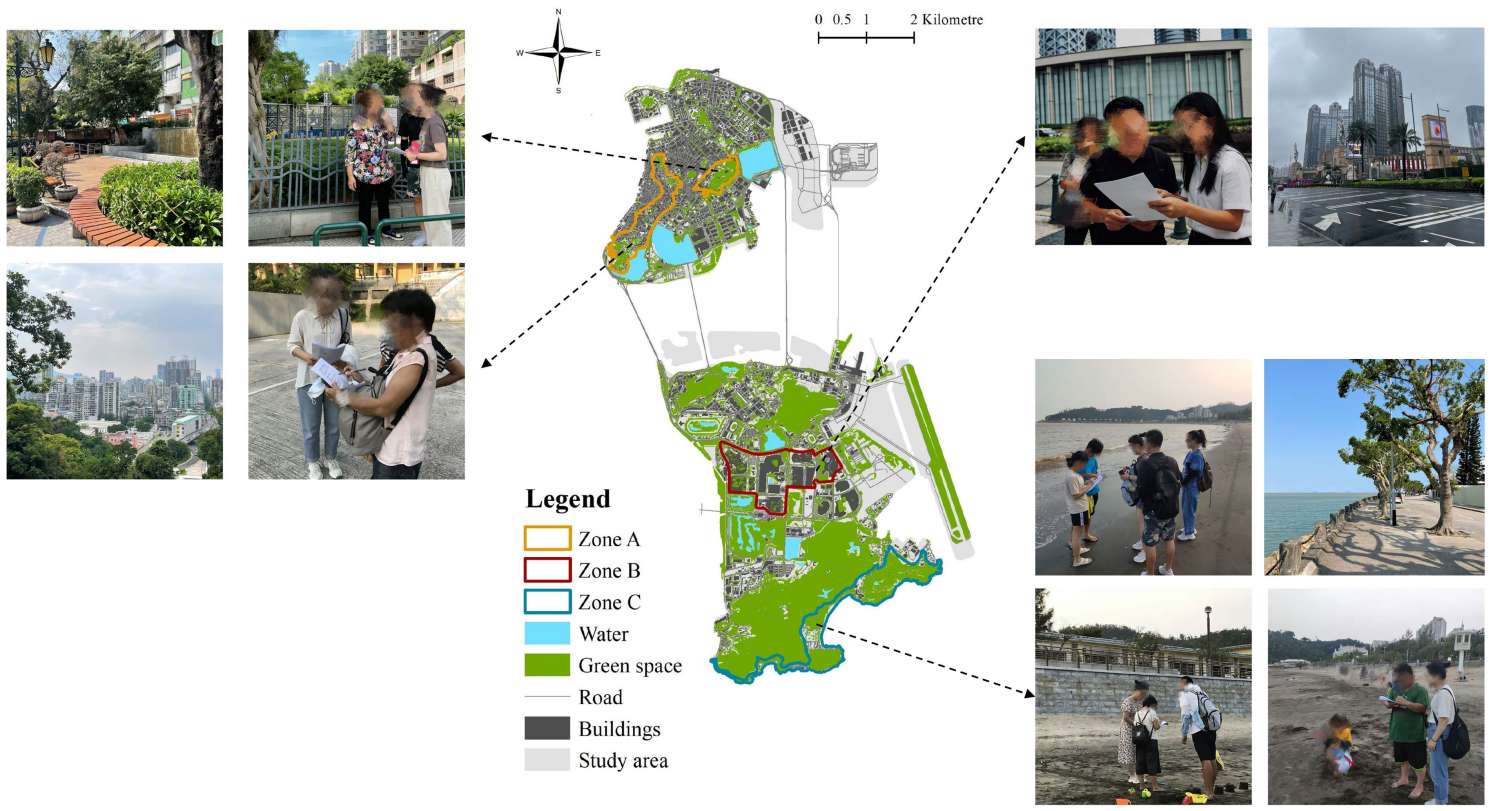
On-site questionnaire distribution and participant engagement.

In parallel, on-site surveys were conducted within the three predefined zones. Trained research assistants employed an intercept survey approach using QR codes to facilitate participation. The exact zone location of each on-site respondent was recorded at the time of data collection, allowing for alignment with zone-level visual indicators. Figure 3 illustrates the on-site survey process, showing the participation of respondents in the survey and the use of QR codes to facilitate data collection. Participation was restricted to non-local tourists aged 18 years or older. Given the absence of a comprehensive sampling frame for transient tourist populations, a non-probabilistic convenience sampling approach was adopted, consistent with common practice in tourism field research (Calder et al., 1981). While this approach limits strict statistical representativeness, it is widely used in urban tourism research where probability sampling of mobile visitor populations is not feasible. Whereas on-site respondents were assigned to zones based on observed survey location, online respondents were classified according to self-reported primary activity zones, and these two procedures may differ slightly in spatial precision. To evaluate potential impacts of this difference, measurement invariance across zones was tested at the configural and metric levels prior to cross-zone comparisons. The results supported acceptable invariance, suggesting that the measurement structure remained stable despite the mixed sampling approach. Nevertheless, minor classification inconsistencies may remain and are acknowledged in the interpretation of zone-level comparisons. All survey procedures complied with ethical standards outlined in the Declaration of Helsinki, and informed consent was obtained from all participants (World Medical Association, 2013).

### 2.3 Street view imagery and visual quantification

To objectively characterize the visual environment of the study area, street view imagery (SVI) was collected using the Baidu Maps Street View API. Images were sampled at approximately 30-meter intervals along the road network within each tourism zone to ensure comprehensive spatial coverage. This procedure resulted in 412 sampling points in the Historic Heritage Zone, 538 in the Modern Entertainment Zone, and 367 in the Coastal and Natural Zone, yielding a total of 1,317 valid sampling locations. Image retrieval was conducted between January and March 2024. The Baidu Street View platform does not provide publicly accessible information on image capture dates, seasons, or time-of-day. As a result, precise temporal metadata are unavailable.

Given the inherent limitations of single-perspective street view imagery, four directional images (0°, 90°, 180°, and 270° headings) were retrieved at each sampling location. Visual indicators were calculated for each direction and then averaged to provide a more comprehensive representation of pedestrian-scale visual conditions. This procedure approximates the pedestrian visual field more closely and reduces potential directional bias. All images were manually screened prior to analysis to exclude night scenes, severely occluded views, and blurred captures. Images displaying substantial temporary obstruction were also removed to minimize distortion in pixel-based segmentation and indicator calculation.

Semantic segmentation of the street view images was performed using the DeepLab-V3+ model, which was pre-trained on the Cityscapes dataset and subsequently fine-tuned to better reflect the urban morphology of Macao. The segmentation process classified image pixels into multiple categories, enabling the extraction of visual environmental indicators relevant to human spatial perception. Based on the segmentation outputs, three objective visual metrics were calculated for each sampling location: the Green View Index (GVI), defined as the proportion of pixels classified as vegetation; the Sky View Factor (SVF), defined as the proportion of pixels classified as sky; and the Building Enclosure Index (BEI), defined as the proportion of pixels classified as building structures.

The Green View Index and Sky View Factor capture visible greenery and sky openness at street level, respectively. The Building Enclosure Index (BEI), defined as the proportion of façade pixels within the street-level image, serves as a visual proxy for perceived enclosure. It does not capture geometric enclosure measures such as height-to-width ratios or three-dimensional morphological configurations commonly discussed in urban design literature (Ewing and Handy, 2009). Accordingly, enclosure in this study refers specifically to façade dominance within the visual field rather than a comprehensive morphological enclosure construct.

These indicators were selected because they capture key aspects of visual openness, enclosure, and natural presence that are closely related to environmental perception in urban settings. Zone-level averages of GVI, SVF, and BEI were computed to characterize the overall visual conditions of each functional zone. Although the survey was conducted over a 12-month period, the visual indicators primarily reflect relatively stable structural characteristics of the built environment, such as façade dominance, sky visibility, and overall spatial enclosure. Seasonal variation may influence vegetation density to some extent; however, no systematic seasonal imbalance across zones was evident in the metadata. Accordingly, the visual indicators are interpreted as representations of prevailing visual conditions rather than time-specific environmental states.

### 2.4 Data analysis

Data analysis followed a two-stage analytical strategy. First, survey data were analyzed using covariance-based structural equation modeling (CB-SEM) to examine relationships among latent psychological constructs. Following the two-step procedure recommended by Anderson and Gerbing (1988), confirmatory factor analysis (CFA) was conducted to assess the measurement model in terms of reliability and validity. Internal consistency reliability was evaluated using Cronbach’s alpha, while convergent validity was assessed using composite reliability (CR) and average variance extracted (AVE) (Fornell and Larcker, 1981; Hair et al., 2019).

The structural model was then estimated using the lavaan package in *R* (version 4.3) (Rosseel, 2012). The significance of indirect effects involving satisfaction and attitude was tested using bias-corrected bootstrapping with 5,000 resamples (Preacher and Hayes, 2008), and model fit was evaluated using commonly accepted indices, including the Comparative Fit Index (CFI) and the Root Mean Square Error of Approximation (RMSEA), based on established guidelines (Hu and Bentler, 1999).

Prior to comparing latent construct patterns across zones, it should be noted that the study did not conduct formal multi-group invariance testing. Accordingly, cross-zone comparisons are interpreted descriptively rather than as strict statistical tests of latent mean differences. The purpose of the zone-level comparison is to provide contextual interpretation of spatial variation in tourists’ psychological evaluations, rather than to support formal multi-group inference.

In parallel with individual-level behavioral modeling, a zone-level descriptive comparison was conducted to situate tourists’ psychological evaluations within different visual environmental contexts. Street-level visual indicators—including the Green View Index (GVI), Sky View Factor (SVF), and Building Enclosure Index (BEI)—were aggregated at the functional zone level to characterize overall visual conditions across tourism zones. The use of street view–derived indicators to represent pedestrian-scale visual environments is well established in urban and environmental research (Li et al., 2015; Chen et al., 2012). These indicators were not incorporated into the structural equation model as individual-level predictors, but were instead used to provide contextual reference for interpreting patterns observed in the behavioral model.

Mean latent construct scores derived from the SEM (e.g., perceived risk, destination image, and satisfaction) were examined across zones to assess whether systematic differences in psychological evaluations co-occurred with variations in visual environmental conditions. Given the limited number of functional zones (*n* = 3), this comparison is descriptive in nature and does not support formal statistical inference. Correlation coefficients are therefore reported solely to summarize zone-level patterns of alignment, rather than to support statistical generalization (Hair et al., 2022).

Accordingly, the zone-level analysis is intended to provide contextual interpretation of spatial variation in tourists’ evaluations, rather than to estimate individual-level environmental effects.

## 3 Result

### 3.1 Descriptive statistics

After data screening, a total of 519 valid responses were retained for analysis. This sample size exceeds commonly recommended thresholds for covariance-based structural equation modeling (CB-SEM), providing adequate statistical power and estimation stability (Hair et al., 2022). The sample exhibited a balanced gender distribution (50.3% male and 49.7% female). Most respondents were young to middle-aged adults, with 58.1% aged between 25 and 45 years. This age structure is consistent with recent post-pandemic tourism patterns, in which younger cohorts demonstrate relatively higher travel mobility (Li et al., 2021). Detailed sociodemographic characteristics of the sample are summarized in Table 1.

**Table 1:** Sample demographics (*N* = 519).

Category	Subcategory	Frequency	Percentage
Gender	Male	261	50.30%
	Female	258	49.70%
Age	18–24 years	65	12.50%
	25–45 years	301	58.10%
	46–60 years	118	22.70%
	Above 60 years	35	6.70%
Education	High school/Vocational	208	40.00%
	Bachelor’s degree	234	45.00%
	Master’s degree or above	77	15.00%
Monthly income (MOP)	Below 5,000	103	19.85
	5,000–10,000	182	35.07
	Above 10,000	234	45.08

Regarding educational background, approximately 60% of respondents reported holding a bachelor’s degree or higher, indicating a relatively well-educated sample. Prior research suggests that higher educational attainment is often associated with greater environmental awareness and pro-environmental orientation (Wang et al., 2021). However, education was not modeled as a predictor in the present study, and this observation is reported descriptively. In terms of income, 45.1% of respondents reported a monthly income above 10,000 MOP, (approximately USD 1,250 or EUR 1,150 based on average 2024 exchange rates). According to official statistics from the Macao Statistics and Census Service, the median monthly income in Macao is approximately 15,000–17,000 MOP, with the average around 20,000 MOP (DSEC (Direcção dos Serviços de Estatística e Censos), 2023). This suggests that the sample includes a substantial proportion of middle-income or higher visitors.

### 3.2 Visual environmental characteristics of tourism zones

**Figure 4:**
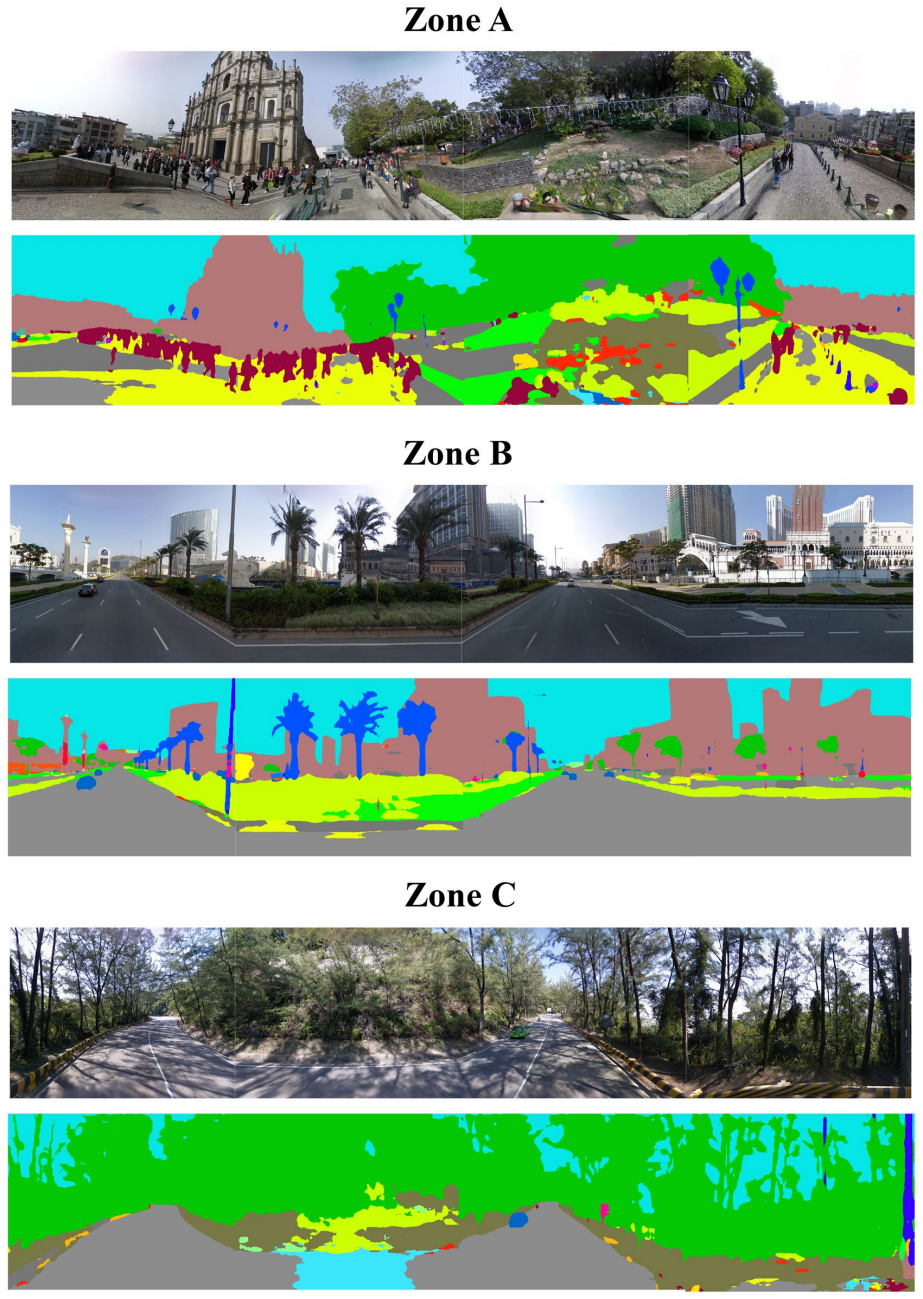
Visualization of semantic segmentation results across three zones using DeepLab-V3+ .

Semantic segmentation of street view imagery was performed to classify each image pixel into predefined categories (e.g., vegetation, sky, building). Figure 4 presents representative segmentation outputs, illustrating how street-level images are decomposed into semantic classes. Based on the segmentation results, pixel proportions corresponding to vegetation, sky, and building façades were calculated for each directional image and then averaged at each sampling location. These proportions were operationalized as the Green View Index (GVI), Sky View Factor (SVF), and Building Enclosure Index (BEI), respectively. Zone-level averages were subsequently computed by aggregating sampling-point values within each tourism zone. The mean values and standard deviations of GVI, SVF, and BEI across the three zones are reported in Table 2. One-way analysis of variance (ANOVA) was conducted to examine whether these aggregated visual indicators differed significantly across zones (*p* < 0.001).

**Table 2:** Objective visual environmental metrics by zone (mean ± SD).

Visual metric	Description	Zone A (Historic)	Zone B (Gaming)	Zone C (Coastal)	*F*-value
Green View Index (GVI)	% of pixels classified as vegetation	15.2% ± 4.1	12.4% ± 3.5	36.8% ± 8.2	124.5***
Sky View Factor (SVF)	% of pixels classified as sky	18.3% ± 5.6	31.5% ± 6.2	45.2% ± 7.9	98.2***
Building Enclosure (BEI)	% of pixels classified as building	42.5% ± 7.3	35.1% ± 6.8	10.4% ± 3.1	115.6***
Road/Pavement	% of pixels classified as road	24.0% ± 4.5	21.0% ± 3.9	7.6% ± 2.2	45.3***

Data derived from semantic segmentation of street view images using DeepLab-V3+.

***p < 0.001 based on ANOVA test.

Zone A (Historic Heritage Zone) exhibited the highest Building Enclosure Index (BEI = 42.5% ± 7.3) and the lowest Sky View Factor (SVF = 18.3% ± 5.6), reflecting a visually enclosed urban environment with limited sky visibility. Vegetation coverage in this zone was relatively low, with a Green View Index (GVI) of 15.2%.

Zone B (Modern Entertainment Zone) was also characterized by a predominantly built environment. Although its BEI (35.1% ± 6.8) was lower than that of Zone A, indicating somewhat wider street profiles, it recorded the lowest GVI among the three zones (12.4% ± 3.5). This visual composition reflects the highly engineered landscapes of integrated resort districts and is consistent with previous studies using similar semantic segmentation approaches in Macao (Shi et al., 2024).

In contrast, Zone C (Coastal and Natural Zone) displayed substantially higher visual openness and natural presence. This zone recorded the highest GVI (36.8% ± 8.2) and SVF (45.2% ± 7.9), alongside a markedly lower BEI (10.4% ± 3.1). These indicators distinguish Zone C from the more densely built urban areas of the Macao Peninsula.

### 3.3 Measurement model assessment

Before estimating the structural model, the measurement model was examined. Model fit was evaluated using several commonly reported goodness-of-fit indices for covariance-based structural equation modeling. The results are presented in Table 3. The *χ^2^*/df value was 2.56, while RMSEA was 0.064. Both CFI (0.94) and TLI (0.92) exceeded the recommended threshold of 0.90, indicating an acceptable level of model fit.

**Table 3:** Model fit indices.

Fit index	Observed value	Threshold
χ^2^/df	2.56	< 3.0
RMSEA	0.064	< 0.08
CFI	0.94	> 0.90
TLI	0.92	> 0.90

The reliability and convergent validity of the constructs were then assessed. As shown in Table 4, composite reliability (CR) values ranged from 0.86 to 0.90, exceeding the minimum recommended level of 0.70. Average variance extracted (AVE) values for all constructs were above 0.50, supporting convergent validity. Standardized factor loadings for individual items ranged from 0.75 to 0.90, with all loadings statistically significant. In addition, given that cross-zone comparisons are intended to provide contextual interpretation rather than formal multi-group inference, the measurement model was estimated for the full sample without conducting formal multi-group invariance testing. Accordingly, subsequent zone-level comparisons should be interpreted descriptively. Based on these results, the measurement properties of the constructs were considered adequate for subsequent structural model estimation.

**Table 4:** Reliability and validity of constructs.

Construct	Items	Factor loading range	CR	AVE
Destination image	5	0.78–0.88	0.89	0.62
Perceived value	4	0.75–0.86	0.87	0.63
Perceived risk	4	0.77–0.85	0.86	0.61
Satisfaction	4	0.80–0.90	0.9	0.69
Attitude	5	0.79–0.87	0.88	0.65
Behavioral intention	4	0.81–0.89	0.89	0.67

CR, composite reliability; AVE, average variance extracted.

### 3.4 Structural model results

**Figure 5:**
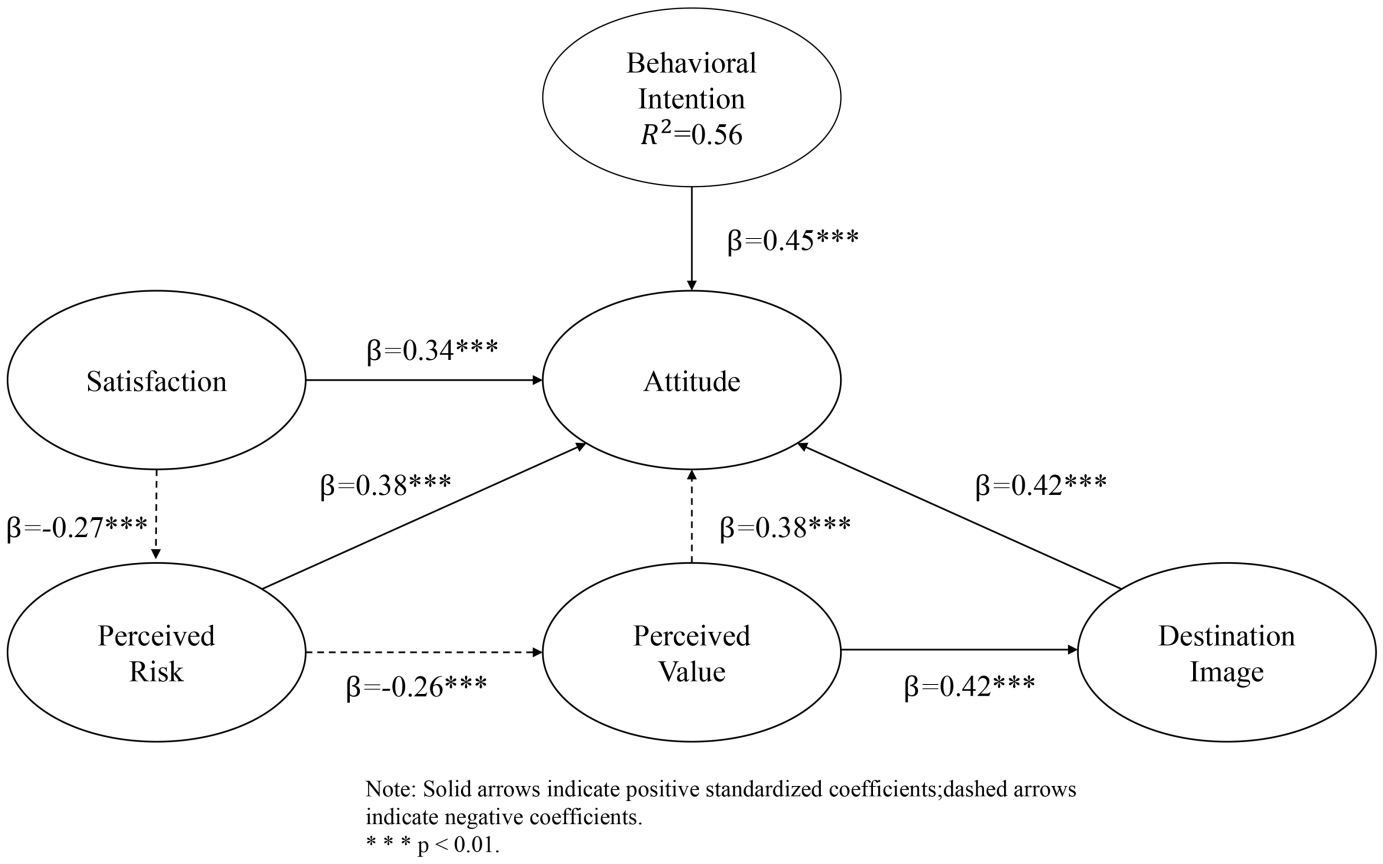
Results of the structural equation model.

After the measurement model was established, the structural model was estimated to examine the associations among the latent constructs. The structural model explained 56% of the variance in responsible tourism behavioral intention (*R^2^* = 0.56). Standardized path coefficients are illustrated in Figure 5. In the figure, solid arrows represent positive associations, whereas dashed arrows denote negative associations. The thickness of each arrow reflects the relative magnitude of the standardized coefficient. Statistical significance levels are indicated by asterisks (*p* < 0.05, *p* < 0.01). This graphical representation allows visual comparison of the relative strength and direction of relationships among the latent constructs within the extended attitudinal framework.

Destination image was positively associated with attitude toward responsible tourism behavior (*β* = 0.42, *p* < 0.01). Perceived value also showed a positive association with attitude (*β* = 0.38, *p* < 0.01). Satisfaction was positively associated with attitude (*β* = 0.34, *p* < 0.01). Attitude, in turn, was positively associated with responsible tourism behavioral intention (*β* = 0.45, *p* < 0.01).

Perceived risk was negatively associated with satisfaction (*β* = −0.27, *p* < 0.01) and attitude (*β* = −0.26, *p* < 0.01). All hypothesized paths were statistically significant in the expected directions. Attitude was positively associated with responsible tourism behavioral intention (β = 0.45, *p* < 0.01) and demonstrated the strongest direct effect on intention among all predictors, reinforcing its central role within the behavioral framework and aligning with theoretical expectations derived from the Theory of Planned Behavior.

**Figure 6:**
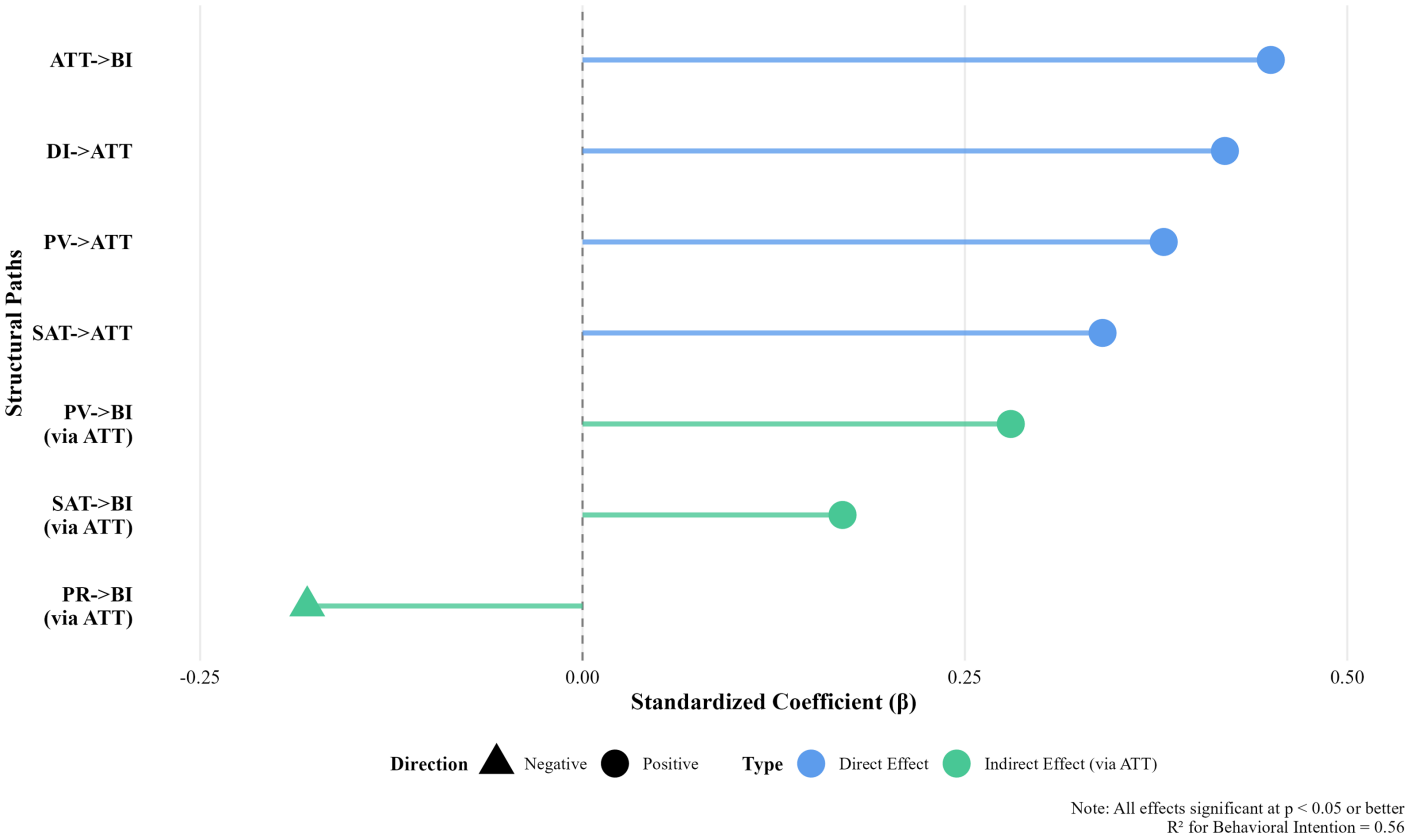
Standardized coefficients from the structural equation model.

Indirect effects were examined using bias-corrected bootstrapping with 5,000 resamples. The results indicate that destination image, perceived value, satisfaction, and perceived risk influence responsible tourism behavioral intention primarily through attitudinal formation. Figure 6 presents the decomposition of direct and indirect effects, showing that the indirect paths transmitted through attitude account for the substantial portion of the total effects. In the case of perceived risk, its influence on behavioral intention operates indirectly through its negative associations with satisfaction and attitude.

Overall, the structural results support a mediation-based interpretation in which cognitive evaluations (destination image and perceived value) and affective responses (satisfaction) shape responsible tourism intention through attitudinal formation, while perceived risk functions as a constraining experiential condition within this pathway.

While the structural model identifies mechanisms operating at the individual level, it does not specify the environmental contexts within which such evaluations emerge. The subsequent zone-level analysis therefore complements the SEM findings by situating these psychological patterns within objectively measured visual environments and provides contextual grounding for interpreting spatial variation in evaluative outcomes.

### 3.5 Alignment between visual environmental contexts and psychological evaluations

To situate tourists’ psychological evaluations within visually distinct urban environments, a zone-level comparison was conducted by aligning aggregated visual environmental indicators with mean latent construct scores derived from the structural equation model. This analysis provides contextual interpretation of spatial variation in evaluations across tourism zones.

Clear contrasts were observed across the three tourism zones. As summarized in Table 5, the Green View Index (GVI) and Sky View Factor (SVF) were positively correlated with destination image (*r* = 0.54 and 0.41, respectively) and satisfaction (*r* = 0.48 and 0.39), whereas the Building Enclosure Index (BEI) showed negative correlations with these constructs (*r* = −0.38 and −0.31). Zones characterized by greater sky visibility and higher levels of visible greenery therefore tended to coincide with more favorable evaluations of destination image and higher levels of satisfaction. In contrast, zones with higher building enclosure and more visually constrained street environments tended to coincide with elevated perceived risk and less favorable destination evaluations. Specifically, BEI was positively correlated with perceived risk (*r* = 0.55), while SVF exhibited a strong negative correlation with perceived risk (*r* = −0.68), suggesting that reduced visual openness coincided with greater congestion-related or spatial discomfort perceptions. At the same time, the correlation between perceived risk and destination image remained moderate (*r* = −0.36), indicating that elevated risk perception did not uniformly eliminate positive destination appraisal.

**Table 5:** Zone-level alignment between visual environmental indicators and psychological constructs (descriptive).

Variables	1	2	3	4	5	6	7
1. Green View Index (GVI)	1.00						
2. Sky View Factor (SVF)	0.62	1.00					
3. Building Enclosure (BEI)	−0.71	−0.85	1.00				
4. Destination Image (DI)	0.54	0.41	−0.38	1.00			
5. Perceived Value (PV)	0.32	0.28	−0.15	0.65	1.00		
6. Perceived Risk (PR)	−0.45	−0.68	0.55	−0.36	−0.22	1.00	
7. Satisfaction (SAT)	0.48	0.39	−0.31	0.58	0.52	−0.27	1.00

Objective visual indicators (GVI, SVF, and BEI) are aggregated at the functional zone level, and psychological constructs represent mean latent variable scores derived from the structural equation model. Values are reported for descriptive comparison only to illustrate patterns of alignment across zones. Given the limited number of zones (*n* = 3), no statistical inference or hypothesis testing is intended.

These patterns are broadly aligned with prior findings in environmental psychology and urban studies, which have documented associations between visual openness, natural elements, and affective appraisal. At the same time, the alignment between visual environmental characteristics and psychological constructs was not uniform across dimensions. For example, perceived value exhibited weaker and less systematic variation across zones than destination image or satisfaction, suggesting that value-related evaluations may depend on a broader set of experiential and symbolic factors beyond immediate visual perception, such as service quality, pricing considerations, or cultural meaning (Rasoolimanesh et al., 2023).

Given that visual indicators were aggregated at the zone level and that only three functional zones were examined, the comparison remains descriptive in scope. The alignment analysis is therefore intended to contextualize spatial variation in tourists’ evaluations rather than to support statistical generalization. For transparency, a detailed zone-level summary illustrating the alignment between visual environmental indicators and psychological constructs is provided in the Table 5. The findings are interpreted as contextual patterns that situate tourists’ psychological evaluations within different urban visual environments. It is also important to clarify the relationship between the destination image construct and the objective visual indicators. Destination image includes perceptual evaluations of the visual and atmospheric qualities of the urban environment. Although this may conceptually relate to objectively measured visual indicators (e.g., GVI, SVF, BEI), the two operate at different analytical levels. Destination image reflects subjective psychological appraisal, whereas the visual indicators represent objective environmental characteristics aggregated at the zone level. The observed alignment should therefore be understood as correspondence between subjective perception and objective spatial context, rather than as mechanical overlap between identical measures.

## 4 Discussion

### 4.1 Psychological correlates of responsible tourism behavior

This study examined responsible tourism behavior in a high-density urban destination using a behavioral framework grounded in the Theory of Planned Behavior. As clarified in the theoretical framework, the model represents a context-specific extension emphasizing the attitudinal pathway, rather than a full replication of the original TPB structure. The results show that destination image, perceived value, and satisfaction are positively associated with tourists’ attitudes toward responsible tourism behavior, whereas perceived risk is negatively associated with both satisfaction and attitude. Attitude was more closely associated with responsible tourism behavioral intention than other psychological constructs, highlighting its role within the behavioral framework.

These findings are consistent with prior tourism research emphasizing the relevance of attitudinal evaluation for pro-social and pro-environmental intentions (Ajzen, 1991; Han, 2015; Ulker-Demirel and Ciftci, 2020). In the structural model, destination image, perceived value, and satisfaction were all significantly associated with attitude, and their effects on responsible tourism behavioral intention were transmitted primarily through this attitudinal pathway. The simultaneous significance of both cognitive evaluations (destination image and perceived value) and affective response (satisfaction) suggests that these components contribute jointly rather than independently to the formation of evaluative orientation. In this respect, responsible tourism intention reflects an integrated assessment of destination attributes and experiential outcomes rather than a response to a single perceptual dimension.

Perceived risk showed a consistent negative association with both satisfaction and attitude. In the present study, perceived risk primarily reflects congestion-related concerns, environmental discomfort, and spatial complexity anxiety within dense urban environments, rather than broader financial or safety-related tourism risks. This pattern is in line with existing tourism research suggesting that risk perception acts as a constraining condition within tourism experiences, particularly in environments characterized by complexity, crowding, or uncertainty (Bae and Chang, 2021; Zheng et al., 2021). In high-density urban destinations, concerns related to safety, congestion, or environmental stress may coincide with lower experiential comfort, which in turn is reflected in less favorable attitudinal evaluations.

### 4.2 Visual environment as a context for psychological evaluation

By incorporating street view–based visual indicators, this study situates tourists’ psychological evaluations within the physical context of dense urban tourism environments. Rather than conceptualizing visual characteristics as direct behavioral drivers, the analysis frames them as contextual conditions that shape the experiential settings in which evaluations are formed. Importantly, visual indicators were not incorporated into the structural equation model as individual-level predictors, and the alignment analysis is descriptive rather than causal. This approach responds to calls in tourism research to better integrate environmental context into behavioral models traditionally dominated by self-reported perceptions (Afshardoost and Eshghi, 2020; Ulker-Demirel and Ciftci, 2020).

The observed alignment between visual openness, greenery, and more favorable psychological evaluations is broadly consistent with findings from environmental psychology and urban design research, which link natural elements and open views to cognitive comfort and affective appraisal (Ulrich, 1984; Hartig et al., 2014; Li et al., 2015). However, given that visual indicators are aggregated at the functional zone level and only three zones are examined, these observations should be interpreted as patterned contextual variation rather than evidence of directional environmental effects.

Similarly, although visually enclosed environments tended to coincide with higher perceived risk, this relationship should not be interpreted as deterministic. As shown in the zone-level comparison (Table 5), the Historic Heritage Zone exhibited relatively higher Building Enclosure Index values alongside elevated perceived risk scores, while still maintaining moderate levels of destination image. Dense heritage districts may simultaneously evoke cultural authenticity and historical significance while also generating spatial discomfort due to congestion or limited visual openness. The positive correlations between visual openness indicators (GVI and SVF) and destination image, combined with the positive association between enclosure (BEI) and perceived risk, suggest that appreciation and discomfort can coexist within the same spatial context rather than forming a simple oppositional pattern. Such environments may therefore give rise to ambivalent evaluations, in which appreciation and perceived risk coexist (Wan and Li, 2013; McCartney, 2020).

These findings highlight the importance of interpreting visual environmental indicators as part of a broader experiential framework. It should also be noted that the destination image scale employed in this study contains visually oriented items, including assessments of streetscape esthetics and architectural impressions. As a result, part of the observed alignment between destination image and street-level visual indicators may reflect conceptual proximity rather than an independent environmental mechanism. In this sense, the visual metrics do not introduce an entirely separate explanatory dimension, but rather provide objective contextual reference for perceptual constructs that are already visually grounded. This distinction is important for avoiding overinterpretation of alignment patterns as novel causal effects.

### 4.3 Implications, limitations, and future research

The findings are particularly relevant for high-density urban destinations characterized by spatial heterogeneity and dual tourism structures, such as Macao, where heritage districts coexist with large-scale entertainment and Modern Entertainment Zone areas (McCartney, 2020). In such settings, responsible tourism initiatives often emphasize communication, education, and behavioral guidance. The present results suggest that these efforts may benefit from greater attention to the spatial and visual conditions in which tourism experiences take place.

In dense heritage areas, higher building enclosure and limited sky visibility were associated with higher perceived risk and lower satisfaction. While this pattern does not imply that visual density directly produces unfavorable evaluations, it indicates that compact spatial configurations may coincide with heightened experiential strain under conditions of visitor concentration. This observation aligns with tourism risk research showing that perceived risk is associated with less favorable attitudes and behavioral intentions in crowded or uncertain environments (Bae and Chang, 2021; Zheng et al., 2021). From a management perspective, responsible tourism strategies in such zones may therefore combine demand-side measures—such as visitor flow management and information provision—with spatial interventions aimed at improving visual comfort, including selective greening or the preservation of visual corridors where feasible.

Conversely, zones characterized by higher greenery visibility and greater visual openness were associated with more positive destination image and higher satisfaction. These findings are consistent with previous studies highlighting the contribution of street-level greenery to perceived environmental quality and experiential well-being (Li et al., 2015), as well as with restoration-oriented research emphasizing the psychological benefits of natural elements in urban environments (Hartig et al., 2014; Ulrich, 1984). For compact destinations, visually open or coastal areas may therefore function as experiential complements to denser urban cores, supporting more diversified tourism experiences without implying uniform behavioral responses across all visitors. Beyond its practical implications, this study also offers methodological insights for tourism research.

From a methodological perspective, this study demonstrates the potential value of integrating objective environmental indicators with survey-based behavioral models in tourism research. Although applications of the Theory of Planned Behavior are well established in tourism studies, reviews have noted that many analyses rely primarily on self-reported perceptions and provide limited spatial or environmental contextualization (Ulker-Demirel and Ciftci, 2020). By incorporating street view–derived visual indicators, this study introduces an empirical dimension that situates psychological constructs such as perceived risk and destination image within their observable physical settings. The operationalization of street-level greenery through the Green View Index follows established practice in urban research (Li et al., 2015), while the inclusion of sky openness and building enclosure aligns with urban morphology studies of street canyon environments (Chen et al., 2012). Together, these indicators provide a transparent and replicable approach to characterizing visual conditions relevant to tourists’ on-site experiences, particularly in high-density urban destinations where spatial variation occurs at fine scales.

Several limitations should nevertheless be considered when interpreting the findings. First, visual indicators were aggregated at the functional zone level and therefore reflect general spatial patterns rather than individual-level exposure or movement within destinations. Second, the street view imagery retrieved via the Baidu Maps Street View API does not provide publicly accessible information on image capture dates, seasons, or time-of-day. Although the selected indicators primarily describe relatively stable structural characteristics of the built environment, seasonal variation—particularly in vegetation—and potential temporal misalignment with the survey period cannot be entirely excluded. In addition, measurement invariance across zones was not formally tested, which may limit strict comparability of latent constructs across spatial groups.

Future research could extend this framework by incorporating longitudinal data, individual mobility tracking, or multilevel modeling to better examine how environmental exposure interacts with attitudinal development over time. Expanding the analytical scope to include additional sensory dimensions—such as soundscape, thermal comfort, or perceived crowding intensity—may further enrich understanding of how complex urban environments shape responsible tourism behavior.

## 5 Conclusion

This study examined responsible tourism behavior in a high-density urban destination by integrating survey-based behavioral modeling with objective measurements of the street-level visual environment. Using Macao as a case study, the analysis explored how experiential evaluations relate to responsible tourism behavioral intention and how these psychological patterns correspond to variation in urban visual contexts.

The findings indicate that cognitive evaluations (destination image and perceived value) and affective responses (satisfaction) contribute to responsible tourism intention primarily through attitudinal formation, while perceived risk operates as a constraining experiential condition that weakens positive evaluative orientation. Attitude emerges as the most proximate predictor of responsible tourism behavioral intention, highlighting its central role within the extended behavioral framework. These results reinforce the importance of attitudinal processes in shaping responsible tourism behavior while situating them within the distinctive spatial conditions of dense urban destinations.

By combining structural equation modeling with street-level visual quantification, this study offers a contextualized extension of behavioral research in tourism. Methodologically, it demonstrates how subjective psychological constructs can be interpreted alongside objectively measured environmental characteristics. Substantively, it underscores the importance of considering urban spatial and visual contexts when examining responsible tourism behavior in compact, heterogeneous destinations. Future research may build on this framework through multilevel or longitudinal designs and by incorporating additional environmental dimensions to further clarify how urban settings interact with psychological processes in shaping tourism-related behavior.

## Data Availability

The original contributions presented in the study are included in the article/Supplementary material, further inquiries can be directed to the corresponding author.
