# Supplementary material for: Exploring the relationship between urban visual density and responsible tourism behavior: a multimodal study of Macao
Source: Front Psychol. 2026 Mar 26;17:1783451. doi: 10.3389/fpsyg.2026.1783451 (PMC13061721; doi:10.3389/fpsyg.2026.1783451)
Supplement: Supplementary file 1 [file Table_1.docx]

Supplementary Material

# Supplementary Data

**Section A: Screening and Travel Profile Please recall your recent trip to Macao and answer based on your actual experience.**

Q1. Which area did you spend the most time in during your visit? (Please select one)

□ Zone A: Historic Centre (e.g., Ruins of St. Paul's, Senate Square)

□ Zone B: Modern Entertainment District (e.g., Cotai Strip, large resorts)

□ Zone C: Coastal and Natural Areas (e.g., Coloane, Hac Sa Beach)

**Section B: Measurement Scales**

*Please indicate your level of agreement with the following statements regarding your experience in the chosen zone (1 = Strongly Disagree, 5 = Strongly Agree).*

| **Construct** | **Code** | **Measurement Items** | **Score (1-5)** |
| --- | --- | --- | --- |
| **Destination Image (DI)** | DI1 | The visual environment of this area is unique and distinctive. |  |
|  | DI2 | The streetscapes here are aesthetically pleasing. |  |
|  | DI3 | The area has a clear and recognizable image. |  |
|  | DI4 | The architectural style (historic or modern) is attractive. |  |
|  | DI5 | The overall atmosphere makes a strong impression on me. |  |
| **Perceived Value (PV)** | PV1 | The experience in this area was worth the time and effort. |  |
|  | PV2 | The quality of the environment justifies the visit. |  |
|  | PV3 | I received good value for the money spent during my visit. |  |
|  | PV4 | Overall, visiting this area was a valuable experience. |  |
| **Perceived Risk (PR)** | PR1 | I felt worried about the overcrowding in the streets. |  |
|  | PR2 | The high density of people made me feel uncomfortable. |  |
|  | PR3 | I was concerned about safety due to the congestion. |  |
|  | PR4 | The complexity of the environment made me anxious about getting lost. |  |
| **Satisfaction (SAT)** | SAT1 | I am very satisfied with my visit to this area. |  |
|  | SAT2 | The experience exceeded my expectations. |  |
|  | SAT3 | I truly enjoyed my time spent here. |  |
|  | SAT4 | I am happy that I chose to visit this specific zone. |  |
| **Attitude (ATT)** | ATT1 | Being a responsible tourist in Macao is a good idea. |  |
|  | ATT2 | It is important to minimize my negative impact on the environment. |  |
|  | ATT3 | I have a favorable opinion towards protecting local heritage and resources. |  |
|  | ATT4 | Engaging in responsible behavior makes my trip more meaningful. |  |
|  | ATT5 | I support the idea of sustainable tourism in high-density cities. |  |
| **Behavioral Intention (BI)** | BI1 | I intend to perform actions that protect the local environment. |  |
|  | BI2 | I am willing to respect local regulations and cultural norms. |  |
|  | BI3 | I will try to avoid disturbing local residents or other tourists. |  |
|  | BI4 | I plan to recommend responsible travel behaviors to others visiting Macao. |  |

**Section C: Demographic Information**

*Please select the option that best describes you.*

**D1. Gender**

□ Male □ Female

**D2. Age Group**

□ 18–24 years □ 25–45 years □ 46–60 years □ Above 60 years

**D3. Educational Level**

□ High school / Vocational school □ Bachelor’s degree □ Master’s degree or above

**D4. Monthly Income (MOP/CNY)**

□ Below 5,000 □ 5,000 – 10,000 □ Above 10,000
